# A model for spatially periodic firing in the hippocampal formation based on interacting excitatory and inhibitory plasticity

**DOI:** 10.1186/1471-2202-16-S1-O6

**Published:** 2015-12-18

**Authors:** Simon N Weber, Henning Sprekeler

**Affiliations:** 1 Technische Universität Berlin, 10587, Berlin, Germany; 2 Bernstein Center for Computational Neuroscience, 10115, Berlin, Germany

## 

Neurons in the hippocampal formation exhibit a variety of spatially tuned firing patterns. The mechanisms by which these different patterns emerge are not fully resolved, although competing computational models exist for several of them. Here we present a new model that can generate all observed spatial firing patterns by a single mechanism. The model consists of a feedforward network with a single output neuron. Its essential ingredients are i) spatially tuned excitatory and inhibitory inputs [e.g., 1] and ii) interacting excitatory and inhibitory Hebbian plasticity. The inhibitory plasticity homeostatically controls the output firing rate by balancing excitation and inhibition [2]. We show in simulations and by a mathematical analysis that the output neuron develops periodic firing patterns along a stimulus dimension if inhibitory inputs are more broadly tuned than excitatory inputs along this dimension. More generally, depending on the relative spatial auto-correlation length of the excitatory and inhibitory inputs, the model exhibits firing patterns that are similar to those of place cells, grid cells (see Figure 1) or band cells (neurons that fire on spatially periodic bands [3]). For inputs with combined spatial and head direction tuning, the same mechanism leads to output firing patterns reminiscent of head direction cells and conjunctive cells (neurons that fire like grid cells in space but only at a particular head direction). A linear stability analysis of the homogeneous steady state accurately predicts the spatial periodicity obtained from simulations. The model combines the robust pattern formation of attractor models [e.g., 4], with the spatial (rather than neural) structure formation of models based on synaptic plasticity [5]. In contrast to attractor models [6], our model predicts that the grid spacing should be robust to global modifications in inhibitory synaptic strength, a distinction which could be experimentally verified.

In conclusion, we propose a feedforward network model that generates all known spatial firing patterns in the hippocampal formation through a single self-organizing mechanism.

**Figure 1:**
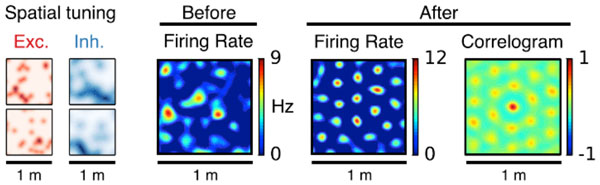
**Example for the emergence of a grid cell**. Columns from left to right: Spatial tuning of excitatory and inhibitory inputs (two examples each); spatial activity pattern of the output neuron before and after learning; auto-correlogram of activity after learning.

## References

[B1] Buetfering C Allen K Monyer H Parvalbumin interneurons provide grid cell-driven recurrent inhibition in the medial entorhinal cortex Nature Neurosci 2014 17 5 710 718 24705183 10.1038/nn.3696

[B2] Vogels TP Sprekeler H Zenke F Clopath C Gerstner W Inhibitory plasticity balances excitation and inhibition in sensory pathways and memory networks Science 2011 334 6062 1569 1573 22075724 10.1126/science.1211095

[B3] Krupic J Burgess N O'Keefe J Neural representations of location composed of spatially periodic bands Science 2012 337 6096 853 857 22904012 10.1126/science.1222403 PMC4576732

[B4] Fuhs MC Touretzky DS A spin glass model of path integration in rat medial entorhinal cortex J Neurosci 2006 26 16 4266 4276 16624947 10.1523/JNEUROSCI.4353-05.2006 PMC6674007

[B5] Kropff E Treves A The emergence of grid cells: Intelligent design or just adaptation? Hippocampus 2008 18 12 1256 1269 19021261 10.1002/hipo.20520

[B6] Couey JJ Witoelar A Zhang SJ Zheng K Ye J Dunn B Recurrent inhibitory circuitry as a mechanism for grid formation Nature Neurosci 2013 16 3 318 324 23334580 10.1038/nn.3310

